# Liposarcoma: A Journey into a Rare Tumor’s Epidemiology, Diagnosis, Pathophysiology, and Limitations of Current Therapies

**DOI:** 10.3390/cancers16223858

**Published:** 2024-11-18

**Authors:** Emily Jonczak, Julie Grossman, Francesco Alessandrino, Crystal Seldon Taswell, Jaylou M. Velez-Torres, Jonathan Trent

**Affiliations:** 1 Department of Medicine, Division of Hematology and Oncology, University of Miami Miller School of Medicine, Miami, FL 33136, USA; 2 Sylvester Comprehensive Cancer Center, University of Miami Miller School of Medicine, Miami, FL 33136, USA; 3 Department of Surgery, Division of Surgical Oncology, University of Miami Miller School of Medicine, Miami, FL 33136, USA; 4 Department of Radiology, Division of Abdominal Imaging, University of Miami Miller School of Medicine, Miami, FL 33136, USA; 5 Department of Radiation Oncology, University of Miami Miller School of Medicine, Miami, FL 33136, USA; 6 Department of Pathology & Internal Medicine, University of Miami Miller School of Medicine, Miami, FL 33136, USA

**Keywords:** liposarcoma, epidemiology, diagnosis, pathophysiology, pathogenesis

## Abstract

Sarcomas are a heterogeneous group of neoplasms that develop from bone and soft tissue. Approximately 80% of sarcomas affect soft tissue, with liposarcoma being one of the most common types, accounting for approximately 13–20% of all soft-tissue sarcomas. Per the World Health Organization, liposarcoma can be broadly classified into four different subtypes based on histologic examination: well-differentiated liposarcoma (WDLS)/atypical lipomatous tumors (ALT), dedifferentiated liposarcoma (DDLS), myxoid liposarcoma (MLS), and pleomorphic liposarcoma (PLS). WDLS/ALT is the most common liposarcoma subtype, accounting for approximately 31–33% of liposarcomas; DDLS accounts for 20%; MLS accounts for 19%; and PLS, the least common subtype, represents 7–8% of liposarcomas. Sarcoma diagnosis is challenging because of its rarity, intrinsic complexity, and diagnostic technological complexity. Sarcomas are misdiagnosed in approximately 30% of cases, leading to delays in diagnosis and access to appropriate therapy and clinical trials. Furthermore, treatment options are limited for those diagnosed with liposarcoma. This review discusses the epidemiology, pathology, and treatment options currently available for liposarcoma.

## Simple Summary

Sarcomas refer to a broad group of rare tumors that develop from bone and soft tissue. Sarcoma diagnosis is challenging because of its rarity and complexity, leading to misdiagnosis and delays in diagnosis and access to appropriate therapy. Liposarcomas are a common form of sarcoma, and there are four subtypes of liposarcomas: well-differentiated liposarcoma/atypical lipomatous tumors, dedifferentiated liposarcoma, myxoid liposarcoma, and pleomorphic liposarcoma. Treatment options are limited for those diagnosed with liposarcoma. Currently, the mainstay of therapy for primary localized liposarcoma is surgical removal of the tumor. Radiotherapy is commonly used in extremity tumors before surgery but has unproven effectiveness in the retroperitoneum. Chemotherapy sensitivity varies by liposarcoma subtype, further complicating treatment. As such, limited conventional treatment options (e.g., radiotherapy or chemotherapy) remain substantial barriers to care. This review provides information about the epidemiology, pathology, and treatment options currently available for liposarcoma.

## 1. Introduction

### 1.1. Epidemiology 

Sarcomas comprise a heterogeneous group of rare neoplasms that develop from bone and soft tissue. With an incidence of ~7 per 100,000 people, they account for 1% of adult cancer diagnoses and 15% of pediatric cancer diagnoses [1,2]. Approximately 80% of sarcomas affect soft tissue [2], and an estimated ~13,500 new soft-tissue malignancies were expected to be diagnosed in the United States in 2023 [3].

Liposarcomas (LSs) are rare mesenchymal soft-tissue sarcomas that are thought to arise from cells in the lipocyte lineages in soft tissues [4]. LSs account for ~13–20% of all soft-tissue sarcomas and are the most common soft-tissue sarcoma worldwide [4,5]. In the United States, LS incidence is increasing. Data between 2001 and 2016 from the US Surveillance, Epidemiology, and End Result (SEER) program and the combined SEER–National Program of Cancer Registries showed an increase in LS diagnosis by 19%, with an annual increase of 1.43% [6]. The average age at diagnosis is 50 years of age, and the incidence increases with age [4,6]. Men account for most new cases (~60%), and LS is found predominantly in Caucasians [5,6]. There are currently no widely accepted LS-specific risk factors. However, as with other types of cancers, potential risk factors for soft-tissue sarcomas include prior radiation, familial cancer syndromes, lymphatic system damage, and long-term exposure to certain toxic chemicals [4]. 

The World Health Organization classifies over 100 soft-tissue sarcoma subtypes, which are characterized by distinct histologic and molecular profiles and variable clinical behavior [7,8]. It classifies LSs into four subtypes based on histologic findings: well-differentiated LS (WDLS)/atypical lipomatous tumors (ALT), dedifferentiated LS (DDLS), myxoid LS (MLS), and pleomorphic LS (PLS) (Table 1) [5,6,9]. Based on US national surveillance databases, the most common subtype is WDLS/ALT, which accounts for 31–33% of LSs; among the other histologic subtypes, DDLS accounts for 20%, MLS for 19%, and PLS for 7–8% [6]. 

### 1.2. Diagnosis and Pathology

Most LS occurs in the extremities (39–41%) and retroperitoneum (21–22%) [6]. It can differ depending on location, but it typically presents as a large asymptomatic mass, usually found incidentally on imaging [11]. However, abdominal and retroperitoneal tumors can present with abdominal pain, distention, urinary obstruction, or weight loss [4,11,12]. 

The initial diagnostic workup for LS includes cross-sectional imaging, such as magnetic resonance imaging (MRI) or computed tomography (CT) [11]. In addition to imaging, clinical guidelines recommend sarcoma diagnosis based on histologic examination, pref-erably by an experienced sarcoma pathologist [9]. A core needle biopsy is the preferred method for obtaining a sample for histologic evaluation, as it is more accurate for defining grade and histologic subtype than fine needle aspiration [11]. Furthermore, the integration of histopathology, immunohistochemistry (IHC), and molecular testing is essential for accurate diagnosis and in guiding clinical management. Recently, genetic testing has helped modify the diagnostic workup for LS to facilitate the diagnosis of subtypes [13,14]. For example, WDLS/DDLS tumors frequently exhibit genetic amplifications in *MDM2*, *CDK4*, and *HMGA2*, while MLS usually presents with *DDIT3-FUS* translocations or, less frequently, *EWSR1-DDIT3* fusions [10]. Following the initial diagnosis of LS, additional imaging can determine the extent of disease and any potential metastases [11].

### 1.3. Diagnostic Challenges 

Sarcomas are initially misdiagnosed in ~30% of cases, leading to delays in appropriate treatment [15,16]. Sarcoma diagnosis is challenging because of its rarity, intrinsic heterogeneity, and the technological diagnostic complexity [15]. For retroperitoneal and intra-abdominal masses, differential diagnoses can include fat necrosis and a large number of tumor types, including leiomyosarcoma, lipoma, schwannoma, undifferentiated pleomorphic sarcoma, fibrosarcoma, angiomyolipoma, myelolipoma, and LS [17,18]. Further complicating the differential diagnosis, intra-abdominal masses may indicate metastasis and not the primary tumor [19]. 

Ancillary diagnostic testing, such as IHC and molecular genetic testing, plays a crucial role in differentiating LS subtypes, particularly when histologic findings are inconclusive [13,14]. IHC helps detect specific protein markers [14], while genetic testing identifies subtype-specific genetic alterations [13]. Importantly, genomic profiling along with detailed histologic examination may help reduce misdiagnoses of LS subtypes [13]. For example, in a study of 384 patients with sarcoma, genetic testing helped refine the pathologic diagnosis in ≤13% of cases [20]. Furthermore, another study that included genetic testing of over 7000 patients found that one-third had potentially actionable genetic mutations [21]. By improving diagnostic accuracy, ancillary diagnostics play a critical role in guiding clinicians toward more targeted and effective treatment strategies, ultimately leading to better patient outcomes [14]. However, genomic testing rates are low, especially in the community setting [21,22]. 

### 1.4. Treatment

Clinical guidelines recommend multidisciplinary team management, including pathologists, surgical oncologists, medical oncologists, and radiation oncologists with expertise in sarcoma diagnosis and therapy [9,16]. In the United States, there are few centers specialized in diagnosing and treating patients with sarcoma [22]. Patients with soft-tissue sarcoma treated at these expert centers have improved clinical outcomes (median overall survival [OS] of 76.2 vs. 64.2 months in high-volume vs. low-volume centers, respectively) [22,23]. However, the limited number of high-volume centers (defined as performing > 10 primary retroperitoneal sarcoma resections per year) may limit patient access and optimal patient care [22,24]. 

For soft-tissue sarcomas, clinical guidelines recommend surgery as a treatment option for resectable disease to obtain complete tumor resections with widely negative margins[9]. As resection is more challenging for some tumors depending on tumor stage, location, and involvement of critical organs, neoadjuvant and/or adjuvant systemic therapy and/or radiotherapy may be used in conjunction with surgery [25]. Radiotherapy improves clinical outcomes in patients with high-grade soft-tissue sarcoma of the extremities [25,26]. For soft-tissue sarcomas of the extremity and trunk, clinical guidelines recommend preoperative radiotherapy (50–50.4 Gy delivered in 1.8–2 Gy fractions), with studies showing reduction in late toxicities, such as subcutaneous fibrosis, edema, and joint stiffness compared with the higher doses (>64 Gy) and volumes associated with postoperative radiotherapy [9]. Results from the DOREMY trial involving patients with extremity or trunk MLS demonstrated that a preoperative radiotherapy dose reduction of 50–36 Gy had similar efficacy with a more favorable toxicity profile [27]. Preoperative hypofractionated radiation or conventional fractionated radiation have shown similar OS results [28]. For sarcomas of the retroperitoneum, preoperative radiotherapy may be considered for patients with high risk of local recurrence [9]. This approach was investigated in the STRASS trial, in which 266 patients with operable, localized, retroperitoneal soft-tissue sarcoma were randomized to either preoperative radiotherapy (50.4 Gy in 1.8 Gy fractions) followed by surgical resection, or surgery alone [29]. The primary endpoint was abdominal recurrence-free survival (RFS), a composite measure that included local recurrence or distant metastasis during preoperative radiotherapy, development of inoperability (a score of 3 on the American Society of Anesthesiologist scale), R2 resection, sarcomatosis, or local recurrence following R0/R1 resection. After a median follow-up of 43 months, the median abdominal RFS was 4.5 years (95% confidence interval [CI]: 3.9–not estimable [NE]) in the radiotherapy-plus-surgery group and 5.0 years (95% CI: 3.4–NE) in the surgery-alone group (hazard ratio [HR] 1.01; 95% CI: 0.71–1.44; log-rank *p* = 0.95). There was no significant difference in abdominal RFS and OS between the groups. In a post-hoc subanalysis of patients with LS, the HR was 0.62 (95% CI: 0.38–1.02); however, the trial was not powered to assess differences by subtype. The authors concluded that preoperative radiation did not improve abdominal RFS. This trial has faced criticism, primarily regarding the challenges in validating the endpoint and issues with radiation planning [30]. Notably, pooled results from the STRASS and STREXIT trials indicated that preoperative radiation was associated with improved abdominal RFS in patients with primary localized retroperitoneal LS, particularly WDLS, though no difference in OS was observed [31]. In contrast, adjuvant radiotherapy for retroperitoneal tumors offers no clear clinical benefit and is not recommended by clinical guidelines [9,25].

The goal of neoadjuvant and adjuvant chemotherapy is to eliminate micrometastatic disease, decrease local recurrence rates, and improve OS [32,33]. Neoadjuvant chemotherapy may also downstage a tumor for organ-sparing surgery and/or help determine individual tumor chemosensitivity, which varies by LS subtype [11,33]. In our clinical practice, adjuvant chemotherapy is usually administered in patients with intermediate or high-grade DDLS, MLS, and PLS tumors that are ≥5 cm (longest diameter) in the extremities. This approach involves evidence- and experience-based shared decision making with the patient and is further supported by a large meta-analysis demonstrating statistically significant improvements in local recurrence, metastasis, and OS [34]. 

Anthracycline-based combination regimens (e.g., doxorubicin or epirubicin with ifosfamide and/or dacarbazine) are widely used first-line therapies for patients with advanced, unresectable, or metastatic soft-tissue sarcomas (Table 2) [9].

Although a randomized phase 3 study of patients with advanced or metastatic soft-tissue sarcomas showed no significant difference in OS between doxorubicin plus ifosfamide and doxorubicin alone (14.3 months vs. 12.8 months, respectively), the combination treatment resulted in significantly longer progression-free survival (PFS) (7.4 months vs. 4.6 months, respectively) and higher OR (26% vs. 14%, respectively) [35]. However, because overall toxicity was more common with the combination therapy (e.g., leukopenia [43%] and neutropenia [42%] being the most common grade 3 and 4 adverse events), doxorubicin plus ifosfamide should only be considered when tumor shrinkage is the goal, particularly to relieve acute symptoms or as neoadjuvant therapy [35]. Nonetheless, we recommend combined doxorubicin plus ifosfamide in patients with LP who have good performance status and require improved response rates. The recommendation is also supported by a retrospective study in patients with retroperitoneal DDLS where a 24% ORR was observed with first-line doxorubicin-based combination regimens, compared to 0% in patients receiving single-agent chemotherapy or gemcitabine plus docetaxel [36]. Given these data and our clinical experience, the frontline use of doxorubicin plus ifosfamide is recommended in the metastatic and adjuvant settings. While we generally do not participate in clinical trials with single-agent doxorubicin to avoid undertreating patients, we occasionally use single-agent doxorubicin in patients with DDLS who have very poor performance status and may not be candidates for combination chemotherapy. 

Long-term outcomes after resection of retroperitoneal sarcomas are generally poor, with 5-year locoregional recurrence at >50% after primary tumor resection for LS in general, and over 80% at 3 years in DDLS [11,37]. Median survival of patients with advanced disease is 12–15 months, and 5-year survival rates have not changed significantly since the 1980s [38,39]. Furthermore, doxorubicin may have hematologic toxicities, including neutropenia, leukopenia, febrile neutropenia, anemia, and thrombocytopenia, and other common toxicities associated with chemotherapy include alopecia, fatigue, and nausea [35,40,41]. Doxorubicin is also associated with cardiomyopathy, a rare long-term complication [42]. These toxicities led to only ~50% of patients completing all six treatment cycles in the phase 3 EORTC 62012 trial [35]. 

## 2. LS Subtypes: Pathogenesis, Clinical Behavior, and Treatment Options

### 2.1. Well-Differentiated LS/Atypical Lipomatous Tumor 

WDLS/ALT is the most common subtype of LS, accounting for ~40–45% of all LS cases [4,10,43]. WDLS/ALT frequently occurs in the retroperitoneum and proximal extremities, often presenting as a slow-growing, painless mass [10,44]. The WDLS/ALT subtype is more common in middle-aged and older adults [12]. WDLS/ALT is a locally aggressive neoplasm composed of mature adipocytes varying in size and display with focal nuclear atypia. Scattered, hyperchromatic, multinucleated stromal cells and monovacuolated or multivacuolated lipoblasts may also be present [45]. ALT and WDLS describe lesions that are identical in morphology and karyotype. However, ALT has historically been used to describe lesions that arise in surgically amenable locations, such as the extremities or superficial trunk, where a wide excision allows removal of the lesion, and the designation “sarcoma” is not warranted. Meanwhile, the term WDLS usually refers to lesions in sites, such as the retroperitoneum and mediastinum, where obtaining wide surgical excision margins is difficult, usually leading to local tumor recurrence, dedifferentiation and potential metastasis or death [45]. On CT and MRI, WDLS/ALT tumors mostly comprise fatty tissue, sometimes showing thick septations or soft-tissue nodules < 1 cm [46]. 

Tumor location is an important prognostic factor and is the main predictor of recurrence. WDLS has a >40% risk of recurrence in the retroperitoneum and a <2% risk of recurrence in the extremities [43]. WDLS lacks metastatic capacity, and grade 1 WDLS has a high 5-year OS rate of 93% [12]. However, WDLS can dedifferentiate to DDLS, which is more aggressive and has higher risk for local recurrence and metastasis [10,12]. The retroperitoneum is the most common site of dedifferentiation [12,25], and as many as 40% of recurrent lesions within the retroperitoneum may exhibit dedifferentiation [43]. The presence of focal nonlipomatous regions > 1 cm on CT or MRI imaging should raise suspicion of dedifferentiation to DDLS [47]. 

The presence of supernumerary abnormal chromosomes (extrachromosomal rings and/or giant rods) containing amplification from the 12q13-15 region are hallmark genetic alterations of both WDLS/ALT and DDLS [12,43,48]. Frequently amplified genes include *MDM2*, *CDK4*, and *HMGA2* and can be detected using IHC, chromogenic in situ hybridization (CISH), and fluorescence in situ hybridization (FISH) [10,12,49,50]. 

As WDLS/ALT tumors are often localized, slow growing, and chemoinsensitive, surgery remains the mainstay of treatment [10,43], with the goal of margin-negative excision [51]. For extremity WDLS, marginal excision can be acceptable to minimize surgical morbidity. However, for abdominal and retroperitoneal tumor resections, attaining a rim of normal tissue is more difficult, as there is often abutment to large vessels, nerves, or bony structures [52]. Morbidity of the surgery should be considered [51].

Tumors of the retroperitoneum have a high rate of local recurrence. This is hypothesized to be a field defect of the retroperitoneal fat; thus, resection of the retroperitoneal fat should be considered [51,53].

Following WDLS/ALT tumor resection, routine follow-ups for a physical exam and imaging (MRI and/or CT) are recommended. For cases of WDLS of the extremity, abdominal wall, or trunk without dedifferentiation, follow-ups should occur every 6–12 months for 2 years and annually thereafter. For retroperitoneal or intra-abdominal WDLS, follow-ups should occur every 3–6 months for 2–3 years, every 6 months for the next 2 years, and then annually thereafter [9]. Patients with WDLS who develop local recurrence will require additional surgery, as there are currently no systemic therapies approved for WDLS [10,43,44]. 

For WDLS/ALT of the extremities, abdominal wall, and trunk without dedifferentiation, there is currently no standard role for adjuvant/neoadjuvant systemic therapy [10,43]. Otherwise, with evidence of dedifferentiation, WDLS is treated similarly to other soft-tissue sarcomas [9,54]. Clinical trials of targeted therapies, such as *MDM2* or *CDK4* antagonists, are viable options for patients with WDLS/ALT [43]. To our knowledge, no medication has been specifically studied for WDLS alone. However, several trials investigating CDK4 inhibitors have included patients with WDLS. In a phase 2 trial of the CDK4 inhibitor palbociclib at a daily dose of 125 mg, 13 of 60 patients (22%) had WDLS [55]. The study reported a PFS rate at 12 weeks of 57.2%, with a median PFS of 17.9 weeks in the overall population. One patient achieved a complete response. Based on these findings, it may be reasonable to consider the use of CDK4 inhibitors in patients with unresectable WDLS.

### 2.2. Dedifferentiated LS (DDLS) 

DDLS is the second most common subtype, accounting for ~20% of LS in the United States, and most frequently presents in middle-aged and older adults [6,12]. The majority of DDLS cases occur in the retroperitoneum; however, other sites include the extremities, the paratesticular region, and the trunk [10,12,43]. DDLS results from the transition of a WDLS to a nonlipogenic sarcoma of variable histologic grade [54]. Around 90% of DDLS cases occur as a de novo tumor and 10% as a recurrence of a preexisting WDLS [10,56]. Most DDLS cases (90%) have a high-grade sarcoma morphology over a broad morphologic spectrum [54]. They have dedifferentiated areas that have a variety of growth patterns, including, most commonly, spindle cell and pleomorphic patterns and, less frequently, inflammatory, giant cell, round cell, or meningothelial-like patterns. 

On imaging, DDLS is characterized by an adipocytic mass with nonlipomatous elements > 1 cm, with soft-tissue density, fluid density, or mixed density [57,58]. While imaging for DDLS is similar to WDLS, DDLS requires a chest CT scan for staging because of its ability to metastasize [9]. [^18^F]2-fluoro-2-deoxy-d-glucose positron emission tomography/computed tomography (FDG-PET/CT) may be used as a problem-solving tool for equivocal CT findings. It can also be used before surgery in patients with advanced disease to confirm metastases are isolated [59] and before biopsy to identify and target metabolically active DDLS components [9]. Furthermore, multiple prospective and retrospective studies have shown that FDG-PET/CT scans in patients with DDLS provide additional data for grading, staging, prognostication, and response to neoadjuvant treatment [59,60,61].

DDLS cases often have high-level amplifications of chromosome 12 (12q14-15), which includes the *CDK4* and *MDM2* genes along with *CPM*, *HMGA2* (coamplified with *MDM2*) and *SAS*/*TSPAN31* [12]. *MDM2* amplification is a nonspecific feature present in up to 40% of sarcomas; however, *MDM2* is considered to be the main driver gene within the 12q amplicon, and its consistent amplification and overexpression may represent the earliest events in the development of LS [12]. DDLS has additional heterogeneous genetic changes compared with WDLS; in addition to the abnormalities in 12q14-15, there are more amplifications, particularly coamplifications of 6q23 and 1p32 [10,12]. 

DDLS is more clinically aggressive than WDLS, exhibiting high local recurrence and a metastatic rate of 15–20%. In patients with DDLS, the 5-year OS rate is 30%, compared with 90% in patients with WDLS [43]. While DDLS is more chemosensitive than WDLS, surgery still remains the standard primary treatment [9]. The goal of surgery for both extremity and retroperitoneal LSs is complete resection of a single specimen surrounded by a continuous layer of healthy tissue [9,62]. Similar to WDLS, for DDLS of the extremities, surgery should aim for microscopically negative margins [51]. Obtaining truly negative resection margins in the retroperitoneum is harder to achieve, and removal of remaining retroperitoneal fat from the hemiabdomen should be considered. Extended resection of multiple organs, nerves, and vessels may be necessary to attain negative margins during resection of retroperitoneal DDLS [63]. There is ongoing debate around how aggressive surgical resections for retroperitoneal LS should be. Many centers around the world perform “compartmental resections”, which include wider surgical resections incorporating adjacent organs and soft tissues even without overt infiltration [51,63]. In retrospective studies, compartmental resections of retroperitoneal sarcomas were associated with decreased local recurrence, decreased distant metastases, and, in one study with a longer follow-up period, improved survival compared with standard surgery [64,65,66]. Additionally, aggressive compartmental surgery has drawn criticism because of the potential risk of sacrificing healthy organs without tumor invasion. The oncologic benefit of OS versus surgical morbidity and mortality is frequently debated [51]. 

Doxorubicin monotherapy is usually a first-line regimen for patients with unresectable, metastatic soft-tissue sarcoma, but doxorubicin in combination with ifosfamide is also recommended in certain patients (Table 2) [9]. In a retrospective study of first-line chemotherapy in 82 patients with DDLS, there was a partial response (PR) in 21%, stable disease (SD) in 40%, and progression of disease (PD) in 39% of patients. All objective responses (ORs) were in patients receiving combination chemotherapy; in this subset, the clinical benefit rate (complete response [CR]+PR+SD for ≥ 6 months) was 44%, and OR was observed in 24% of patients [36]. Doxorubicin and dacarbazine may benefit patients with renal dysfunction or other comorbidities precluding ifosfamide use [67]. The standard second-line therapy for metastatic disease is gemcitabine (900 mg/m^2^ over 90 min, fixed-dose rate, on days 1 and 8) plus docetaxel (100 mg/m^2^ on day 8 only) every 21 days, resulting in a 32% tumor response (CR+PR+SD for >24 weeks) in patients with recurrent or progressive soft-tissue sarcoma, including DDLS [68]. This recommendation is further supported by a retrospective analysis of 65 patients with recurrent or metastatic WDLS and DDLS on gemcitabine-based therapy, 90.3% of whom were treated with gemcitabine-docetaxel. All patients who showed a response had DDLS. The overall population demonstrated an OR rate of 9.7%, a median PFS of 9.2 months, and a median OS of 18.8 months, suggesting that gemcitabine plus docetaxel is a reasonable second-line option for patients with DDLS [69]. Other notable systemic treatment options available for DDLS include trabectedin (1.5 mg/m^2^ by 24 h infusion every 21 days) and eribulin mesylate (1.4 mg/m^2^ intravenously on days 1 and 8 of 21-day cycles), which are US Food and Drug Administration-approved for patients with unresectable or metastatic LS who have received prior anthracycline-based chemotherapy [70,71]. 

### 2.3. Myxoid LS (MLS) 

MLS accounts for 20–35% of all LS cases and most commonly arises in the proximal extremities [10,56,72]. MLS presents earlier than other LS subtypes, with a peak incidence in young to middle-aged adults, and is the most frequent LS in children and adolescents [56,72].

MLS is clinically and pathologically differentiated from WDLS and DDLS. It is composed of small, uniform nonlipogenic spindle tumor cells and a variable number of scattered lipoblasts, in a richly myxoid stroma containing a prominent plexiform vascular network [10,72]. MLS can be classified as low grade or high grade (formerly round-cell LS). Mitotic activity and necrosis are typically absent in low-grade MLS; accordingly, it is associated with an 80–90% 10-year survival rate [72]. However, high-grade MLS is associated with increased mitotic activity and tumor necrosis. High-grade MLS is defined by the presence of hypercellular areas with minimal cytoplasm composed of round cells containing larger and more hyperchromatic nuclei relative to the primitive spindle cells of low-grade MLS. It is essential to document tumor progression to high-grade/round cell morphology, as tumors with a round cell component of >5% may behave aggressively [10,72], and local recurrence, metastasis, and survival are heavily impacted by the round cell percentage [73]. Kaplan–Meier analysis using SEER data demonstrates that patients with primary MLS have a 5-year survival rate of 76.4%, while the 5-year survival rate for patients with high-grade MLS (having a round cell component) is 54.9% [5]. 

More than 95% of MLS cases are characterized by t(12;16)(q13;p11) translocations, resulting in *FUS-DDIT3* gene fusion [56]. Approximately 5% of MLS tumors harbor a t(12;22)(q13;p12) translocation, resulting in *DDIT3*-*EWSR1* gene fusion on 22q12 [56]. Expression of NY-ESO-1, an immunogenic cancer testis antigen (CTA) whose expression is normally restricted to reproductive cells in adults, shows high-frequency (56–100%) re-expression in MLS [74,75]. Expression of MAGE-A4, another CTA, has a frequency of detection of 0–68% in MLS [76].

The preferred imaging method for MLS is full-body MRI or CT with contrast of the chest/abdomen/pelvis and MRI of the full spine [9,77,78]. Evaluation and surveillance should be done via MRI of the entire spine because of the preponderance of MLS to metastasize to bone and invade the spine [9,10,78]. On imaging, MLS presents as well-defined large lobulated enhancing masses, with fluid-dense components reflecting the myxoid elements [57]. Intralesional fat is not always evident on imaging but, when visible, helps to narrow the differential diagnosis [46]. Round cells, when present, can be identified as contrast-enhancing, nonfatty, nonmyxoid components [79]. Myxoid bone metastases are often not well seen on CT or FDG-PET/CT but can be readily identified on MRI, given their water content [77,80]. As such, whole-body MRI has been proposed for detection in high-risk patients [78,80].

Surgery is the standard primary treatment for MLS, and the same principles of surgery described for DDLS are applicable for MLS [9,62]. Unlike other LS subtypes, MLS is typically chemo- and radiosensitive [10]. In patients with primary nonmetastatic disease, preoperative radiotherapy may improve resectability [27]. In a 2012 retrospective analysis of 37 patients with histologically confirmed MLS, combination doxorubicin and ifosfamide chemotherapy in patients with resectable disease resulted in a PR of 38.5% and SD of 61.5%. In patients with advanced disease, 54.5% achieved PR, and 45.5% had SD [81]. The difference in response between resectable and advanced disease groups was not statistically significant (*p* = 0.48). Median time to progression was 23 months, and OS was 31.1 months in patients with advanced disease, while patients with resectable tumors had a 5-year disease-free survival rate of 90% [81]. Trabectedin has been shown to improve clinical outcomes in all subtypes of LS but can be especially beneficial in MLS [38]. In a long-term retrospective analysis of 32 patients, whose MLS was treated with trabectedin, 14 PRs and 2 CRs were reported with an OR rate (ORR) of 50% (95% CI: 32–68%). An additional 14 patients (44%) had SD with minor tumor shrinkage (>0–<25%), and the disease control rate (CR+PR+SD) was 90% [82]. Although they are actively used in DDLS and PLS, gemcitabine-based chemotherapy regimens have not been thoroughly studied in MLS and are not recommended [9,83]. For patients with local advanced or metastatic MLS, real-world data demonstrate a median 12-month PFS of 37.5% and 12-month OS of 84.6% from the start of first-line therapy; from the start of second-line therapy, median PFS is 3.5 months, and median OS is 25.7 months [84]. Clinical trials evaluating T-cell therapy directed at NY-ESO-1 and MAGE-A4 show promising activity in early studies, and several trials in MLS are underway [85]. 

### 2.4. Pleomorphic LS (PLS) 

PLS, the least common subtype, represents 5% of LS cases and is one of the most clinically aggressive LS subtypes [54,86]. PLS most commonly presents in older adults and typically arises in the extremities; however, it can arise in the trunk, retroperitoneum, abdominal wall, chest wall, and even the head and neck region [87,88]. When located nonsuperficially, it is usually characterized by progressive growth of a painless mass that is overlooked until other symptoms manifest [87]. 

PLS appears as a high-grade undifferentiated sarcoma with lipoblastic (often pleomorphic) differentiation [10,54,86]. To detect PLS, MRI with contrast is preferred for the extremities, CT with contrast is preferred for retroperitoneal and intra-abdominal sites, and only CT with contrast is recommended for the lungs [9,59]. On CT and MRI, LS appears as a well-circumscribed mass containing little or no fat, showing internal hemorrhage and necrosis [46]. Because PLS has a wide morphologic spectrum, with diverse chromosomal rearrangements and genomic profiles, it can be confused with numerous adipocytic and nonadipocytic neoplasms [54,86]. Traditionally, lipoblast identification was considered sufficient to diagnose PLS; however, recent description of homologous lipoblastic differentiation (mimicking PLS) in DDLS has complicated diagnosis. DDLS and PLS should be distinguished in the diagnostic stage when encountering high-grade sarcoma with lipoblastic differentiations [54]. Of note, the dedifferentiated component of DDLS can appear morphologically as PLS. Recently described and extremely rare myxoid, pleomorphic LS is a subtype of LS commonly found in the mediastinum [89]. 

PLS is not associated with the 12q13-15 amplicon and does not typically show *MDM2* overexpression by IHC, *MDM2* amplification with CISH or FISH, or coexpression of *MDM2* and *CDK4* [12,49,50,54]. Up to 50% of patients have been described as having deletion of 13q14.2-5 (containing *RB1*). Also, *TP53* mutation is commonly observed in patients with PLS, and some patients have been shown to have loss of *NF1* [10]. 

PLS is the most aggressive subtype, with poor overall outcomes compared with other LS subtypes [86]. Surgery to achieve negative margins is the mainstay of treatment, including radical resection for patients with primary disease [9,87]. Radiotherapy and surgery are correlated with improved clinical outcomes [88]. PLS has intermediate chemosensitivity, and there is no statistically significant difference in ORR between anthracycline-containing regimens and non–anthracycline-containing regimens (42% vs. 31%, *p* = 0.5) or between single-agent or combination chemotherapy (42% vs. 35%, *p* = 0.7) [10,90]. Similar to DDLS and MLS, tumors > 5 cm and of high grade are treated with a multidisciplinary approach of chemotherapy, radiotherapy, and surgery [9,69]. Patients with metastatic disease are treated with similar regimens to DDLS in the recommended order of greatest efficacy: doxorubicin plus ifosfamide, gemcitabine plus docetaxel, trabectedin, eribulin and dacarbazine (or temozolomide) [9]. There is a high rate of local recurrence and distant metastases; approximately a third of patients with complete resection develop distant metastasis [86]. A SEER database analysis of 555 patients with PLS found a 5-year survival rate of 54% and a 10-year survival rate of 40%, which are significantly worse than other LS subtypes [88].

## 3. Conclusions

Lack of effective diagnosis and limited tolerable conventional radiotherapy or chemotherapy treatment options for patients with locally advanced or metastatic LS is evident, as median survival for patients with advanced disease is 12–15 months and 5-year survival rates have not changed significantly since the 1980s [15,16,38,39]. Better-tolerated therapies are needed. Promising emerging systemic therapies are under investigation in LS, including *MDM2* antagonists, CDK inhibitors, tyrosine kinase inhibitors, and immunotherapies [10]. 

## Figures and Tables

**Table 1:** Epidemiology and clinical characteristics of liposarcoma subtypes.

Subtype	Well-Differentiated Liposarcoma (WDLS)	Dedifferentiated Liposarcoma (DDLS)	Myxoid Liposarcoma (MLS)	Pleomorphic Liposarcoma (PLS)
**Prevalence (%)**	40–45	20	20–30	5–8
**Age of peak incidence**	Middle-aged and older adults	Middle-aged and older adults, and rare occurrences in children and adolescents	Young to middle-aged adults, children, and adolescents	Middle-aged and older adults
**Morphology**	Composed of lobules of mature adipocytes that vary in size and are subcompartmentalized by thick, irregular fibrous bands	Broad morphologic spectrum. Spindle cell and pleomorphic patterns, inflammatory, giant cell, round cell, or meningothelial-like patterns	Extremely hypocellular, featuring a bland spindle cell proliferation set in an abundant myxoid background. Lipoblasts are most often monovacuolated and cluster around vessels or at the periphery of the lesion. Presence of a thin-walled, capillary-sized vascular network, organized in a distinctive plexiform pattern. High-grade MLS is defined by the presence of hypercellular areas	Presence of lipoblasts
**Imaging**	MRI or CT with intravenous contrast of intra-abdominal or retroperitoneal sites	Consider PET/CT for retroperitoneal/intra-abdominal sites to help differentiate WDLS and DDLS and to determine site for biopsy [9]	MRI of total spine and whole body; MRI or CT with intravenous contrast of abdominal/pelvic sites	MRI or CT with intravenous contrast of intra-abdominal, pelvic, or retroperitoneal sites; CT with contrast of lung
**Genomic**	12q13-15 amplification	12q14-15 amplification with other chromosomal abnormalities (particularly coamplifications of 6q23 and 1p32)	t(12;16)(q13;p11) with *FUS-DDIT3* fusion	Deletion of 13q14.2-5 (containing *RB1*), mutation or loss of *TP53*, loss of *NF1*
**Histologic appearances** **Hematoxylin and eosin stain** **Arrows indicate small lipoblasts with nuclear indentation and vacuolated cytoplasm**	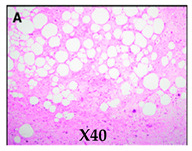	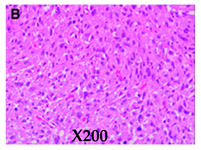	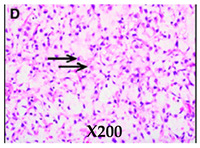	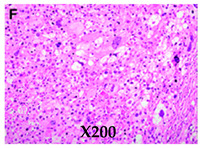

Images adapted with modifications from [10]. DDLS = dedifferentiated liposarcoma; MRI = magnetic resonance imaging; CT = computed tomography; PET/CT = positron emission tomography/computed tomography; WDLS = well-differentiated liposarcoma.

**Table 2:** Authors’ proposed systemic therapy regimens for patients with metastatic or unresectable liposarcoma by subtype and line of therapy *.

Subtype ^†^	First Line	Second Line	Third Line	Fourth Line	Fifth Line
Dedifferentiated	Doxo + ifos ^‡^	Gem + doce	Trabectedin	High-dose ifos ^§^	Eribulin
Myxoid	Doxo + ifos ^‡^	Trabectedin	High-dose ifos	Eribulin	
Pleomorphic	Doxo + ifos ^‡^	Gem + doce	Trabectedin	High-dose ifos	Eribulin

* This table encapsulates the general approach to systemic treatment in the authors’ clinical practice; the order in which second and subsequent lines of therapy are listed here does not necessarily imply priority for these treatment regimens. ^†^ CDK4 and MDM2 antagonists are currently being investigated in clinical trials. ^‡^ Single-agent doxo may be considered as first-line therapy in patients with poor performance status who require palliation of symptoms. ^§^ The high-dose ifos regimen consists of a total of 14 g/m^2^, which may be administered as a 7- to 14-day continuous infusion or 2 g/m^2^ IV bolus every 12 h for 7 doses in the inpatient setting. Doce = docetaxel; doxo = doxorubicin; CDK = cyclin-dependent kinase; gem = gemcitabine; ifos = ifosfamide; IV, intravenous; MDM = murine double minute.

## Data Availability

No new data were created or analyzed in this study.
